# Factor structure and internal reliability of cultural belief scales about colorectal cancer screening among Koreans in the Republic of Korea

**DOI:** 10.1186/s12889-018-6240-9

**Published:** 2018-11-29

**Authors:** Shin-Young Lee

**Affiliations:** Department of Nursing at Chosun University, 309 Pilmun-daero, Dong-gu, Gwangju, 501-759 Republic of Korea

**Keywords:** Colorectal cancer, Culture, Koreans, Beliefs, Instruments

## Abstract

**Background:**

Culturally sensitive, reliable and valid cultural belief scales for colorectal cancer (CRC) screening in Koreans in the Republic of Korea are not available in the literature. The purpose of this study was to adapt and validate existing cultural belief scales for CRC screening in Koreans.

**Methods:**

Individual interviews, expert reviews, and a pilot test were conducted for instrument adaptation, and a cross-sectional survey with 884 Koreans was conducted for instrument validation. Construct validity using exploratory and confirmatory factor analyses and reliability of the Korean version of the instruments were examined.

**Results:**

Exploratory factor analysis using c four factors that accounted for 48.12% of the variance. The validity and reliability of the cultural belief scales were supported by confirmatory factor analysis and Cronbach’s alpha.

**Conclusions:**

The results of the present study showed that the four-factor cultural belief scales were culturally sensitive, reliable and valid in Koreans. The final cultural belief scales could be used to identify cultural beliefs more accurately and specifically, as well as to develop effective interventions to increase CRC screening in Koreans in the Republic of Korea.

**Electronic supplementary material:**

The online version of this article (10.1186/s12889-018-6240-9) contains supplementary material, which is available to authorized users.

## Background

Colorectal cancer (CRC) is the third most commonly diagnosed cancer among Koreans in the Republic of Korea [1]. CRC incidence rates increased significantly in Koreans aged 40 and older, and 90% of patients with CRC were aged 40 and older [2]. Compared with people in Western countries, Koreans aged younger than 50 years old were more likely to have CRC in the Republic of Korea; thus, the Korean National Cancer Committee recommended CRC screening for Koreans aged 45 to 80 [3]. CRC screening rates remain low despite the many opportunities available to Koreans for CRC screening tests by participating in the National Cancer Screening Program that the Korean National Health Insurance Service has provided or by visiting any medical facilities. The rates of CRC screening, including fecal occult blood test and colonoscopy, among Koreans aged 50 and older ranged from only 18.1 to 33.9% in the last 10 years (2007–2016) in the Republic of Korea [4].

A review of the literature revealed that socio-demographic factors (e.g., low income) and beliefs (e.g., barriers) were risk factors Koreans who did not undergo CRC screening [5–7]. Particularly, Korean cultural beliefs, including (a) crisis health orientation toward health care use and (b) external influences on cancer screening behaviors (e.g., familism or fatalism), contributed in part to low CRC screening test utilizations among Korean populations [8–14]. Few studies have performed detailed investigates on how cultural beliefs influenced cancer screening behavior among Koreans in the Republic of Korea. Therefore, we extended the literature review by addressing cultural beliefs and cancer screening among Korean Americans in the US. First, the main barrier to cancer screening was the crisis health orientation toward health care use in Koreans (i.e., Koreans would seek medical care only if they had symptoms) [10, 11]. Similarly, a lack of symptoms was the most common reason for not undergoing cancer screening among Korean Americans [8, 12]. Second, external influences on cancer screening behaviors (e.g., family or fate) were revealed by studies in Korean populations [9, 12–14]. Although family members had encouraged cancer screening, many Koreans [9, 14] and Korean Americans [12, 13] had not sought cancer screening because of a lack of time due to family issues. Additionally, many Korean Americans believed that a life is predetermined since birth due to their traditional cultural beliefs stating that humans cannot control life and death; only God or an unknown supernatural power decides whether we are healthy or ill [12, 13]. These beliefs cause some Korean Americans to ignore the value of cancer screening because they believe they will have cancer regardless of whether they undergo cancer screening [12].

The literature review identified that cultural beliefs importantly influenced cancer screening behaviors, and a Korean version of cultural belief scales for CRC screening was developed by Lee et al. [15] to be culturally sensitive for Korean Americans based on Russell’s theoretical framework [16] combined with both the Cultural Assessment Model for Health [17] and the Powe Fatalism Model [18]. Given that other studies used only a few items to measure cultural beliefs regarding CRC screening behaviors among Korean Americans, Korean cultural belief scales for important cultural constructs, including cancer fatalism, health fatalism, health temporal orientation (crisis or preventive health orientation toward health care use) and personal control (internal or external control), were tested for construct validity and reliability and were found to have good performance for Korean Americans in the US [15]. However, currently, little research has been conducted to develop cultural belief scales for Koreans in the Republic of Korea, although cultural beliefs, such as crisis health orientation, familism, and fatalism, were revealed in cancer screening research with Koreans [9–11, 14]. Culturally sensitive, reliable and valid instruments measuring cultural beliefs are needed to obtain accurate study results, to identify behavioral risk factors and predictors of cancer screening behaviors, and to develop effective appropriate intervention programs to improve low CRC screening utilization among Koreans in the Republic of Korea. To develop reliable and valid cultural belief scales for Koreans, we used the cultural belief scales for Korean Americans [15] because both Korean populations have the same traditional Korean culture. However, constructs and items measuring cultural beliefs regarding CRC screening behaviors may be slightly different between Korean Americans in the US and Koreans in the Republic of Korea, and we needed instrument adaptation processes to make existing cultural belief scales culturally appropriate for Koreans regarding CRC screening behaviors. The purpose of this study was to adapt and validate the cultural belief scales regarding CRC screening behaviors among Koreans in the Republic of Korea.

## Methods

This investigation was comprised of a descriptive qualitative study and a cross-sectional quantitative study through four steps (step 1: individual interviews, step 2: expert reviews, step 3: a pilot test, and step 4: a cross-sectional survey). Steps 1, 2, and 3 were used for instrument adaptation, and step 4 was conducted to test the psychometric properties of the instruments for Koreans in the Republic of Korea. This study’s sample in several steps 1 to 4 consisted of Koreans who were aged 40 years and older.

### Instruments

The initial instruments for cultural beliefs, including cancer fatalism, health fatalism, health temporal orientation, and personal control [15], were adapted, modified, and tested by the author of the present study to measure cultural beliefs about CRC screening while being culturally appropriate for Korean Americans in the US based on the cultural assessment for health [17] and cancer fatalism [18]. In Lee et al.’s study [15], Korean versions of culturally appropriate instruments were developed for Korean Americans, specifically for cancer fatalism (beliefs that death is inevitable when cancer is present), health fatalism (notions of fate, luck, destiny, and predetermination regarding diseases or health conditions), health temporal orientation (perspectives on current health beliefs and behaviors related to concerns about future health) and personal control (ability to plan activities to control or direct factors within the environment). Among the cultural belief constructs, the health temporal orientation scale consisted of items for crisis health orientation (symptoms are cues to seek health care) and preventive health orientation (identifying health problems early is important), and personal control consisted of items for internal control (the power to affect change lies within) and external control (fate, luck and change significantly impact outcomes). The initial Korean version of the cultural belief scales [15] consisted of a total of 52 items: cancer fatalism (15 items), health fatalism (15 items), health temporal orientation (5 items for preventive health orientation and 3 items for crisis health orientation), and personal control (4 items for internal control and 10 items for external control). The initial cultural belief scales established construct validity using exploratory factor analysis (EFA) and confirmatory factor analysis (CFA), and they had good reliability, with Cronbach’s alpha coefficients of 0.77 to 0.88 [15]. All scales were measured on a five-point Likert scale from strongly disagree to strongly agree.

### Step 1: Individual interviews

Individual interviews were conducted to investigate cultural belief concepts, including fatalism, health temporal orientation, and personal control regarding CRC screening, to ensure that the scales that were culturally sensitive and comprehensive for Koreans. The convenience sampling method was used to recruit participants from churches or community centers. A total of 35 Koreans (11 men and 24 women; 20 participants aged 40 to 64 and 15 participants aged 65 and older) participated in individual interviews.

During the interviews, participants discussed cultural beliefs related to CRC screening behavior via semi-structured, open-ended questions (Table 1). Each individual interview was audiorecorded and transcribed verbatim, and the data were coded into concepts. After reviewing the interview transcripts, the principal investigator compared concepts during individual interviews with the cultural belief scales to determine differences between them for instrument adaptation for Koreans.

**Table 1:** Individual interview guide

Concepts	Discussion questions
Cancer fatalism	If people have colorectal cancer, will they die from it regardless of what they do? What do you think of fatalism and cancer?
Health fatalism	How are external controls, such as fate, luck, destiny, and predetermination, associated with diseases or health conditions?
Health temporal orientation	How important do you think it is to detect health problems early? What do you actively do to be healthy in the future?
Perceived control	Who do you think has control over identifying health problems early?

Individual interviews revealed that many Koreans indicated fatalism related to cancer, health and disease, and life and death. The many participants mentioned that cancer was a deadly disease but that it could be treatable if it was found early. Most participants also believed in health fatalism, i.e., that people were born to be healthy or suffer from diseases based on their genes that were inherited from their parents. Regarding health temporal orientation, all of the participants agreed that it is important to detect health problems early, but most of them said that cancer could be prevented by their efforts, such as maintaining a healthy diet and regular exercise, although they did not consider going to medical facilities for cancer screening as a preventive behavior; CRC screening was considered part of the treatment process only when they had abnormal symptoms and disease. Regarding personal control, most of the participants thought that cancer can develop from external forces that cannot be controlled but that people should make efforts to engage in healthy behaviors to prevent cancer. External influences from doctors, family, friends, and other influential individuals enabled them to obtain cancer screening.

All items in the cultural belief scales remained unchanged because Koreans in the Republic of Korea had almost the same cultural beliefs as Korean Americans. Based on two distinct fatalism versions that emerged from the individual interviews, two versions of the fatalism scales (cancer and health fatalism scales) were included in the present study, which were the same as those in the previous study [15]. However, in the present study, of the items in the initial health fatalism scale, three items (“I think it’s fate to get cancer;” “I think cancer is always fatal;” and “I think there is little one can do to prevent cancer”) were moved to the cancer fatalism scale because they were related to cancer based on findings from the individual interviews, and the results of the previous study [15] showed that these three health fatalism items were associated with cancer fatalism.

### Step 2: Expert reviews

Six Korean researchers who were faculty members with doctoral degrees in health-related areas measured the content validity of the cultural belief scales. The experts independently rated content representativeness of each item on a four-point ordinal scale from not relevant to highly relevant. Item-level content validity was calculated by dividing the number of experts who assigned a rating of either 3 or 4 by the total number of experts, and scale-level content validity index/average was calculated by dividing the sum of the scores of item-level content validity by the number of items [19]. All item-level content validity and scale-level content validity values for the cultural belief scales were 1.0 because the experts rated all of the items either 3 or 4. The experts suggested making unclear items clearer and understandable for Koreans, and items were modified accordingly.

### Step 3: A pilot test

The purpose of the pilot test was to identify problems by testing the cultural belief scale with a large sample. Twenty-four participants (10 men and 14 women, 11 participants aged 40 and 64 and 13 participants aged 65 and older), who were recruited by convenience sampling methods from churches and community centers, were included in the pilot test. The participants completed the survey in 20 to 30 min. The participants made suggestions, such as removing redundancies in the survey questionnaire, and the items were modified accordingly.

### Step 4: A cross-sectional survey

#### Sample and data collection

A total of 1025 Koreans were recruited from churches, community centers, stores, apartment communities, and companies in a metropolitan area in the Republic of Korea using convenience and chain referral sampling methods for the cross-sectional survey to assess psychometric properties of the final version of the cultural belief scale for CRC screening utilization among Koreans. Survey questionnaires were distributed in person to Koreans aged 40 and older. A total of 884 individuals participated in this study (response rate = 86.2%). The absolute number of cases and the subject-to-variable ratio were used to determine the minimum sample size in factor analysis. A sample size of more than eight hundred cases in the present study was satisfactory for factor analysis based on a calculation of either the absolute number of cases or the subject-to-variable ratio [20–23].

The cultural belief scales in the questionnaire consist of four sections: cancer fatalism (18 items), health fatalism (12 items), health temporal orientation (3 items for crisis health orientation and 5 items for preventive health orientation), and personal control (4 items for internal control and 10 items for external control) (Additional file 1).

#### Data analysis

For factor analysis of the cultural belief scales, both EFA and CFA were performed using SPSS Version 24 [24] and AMOS 23 [25] in the present study. As complementary methods, EFA was conducted to identify a scale’s underlying structure while CFA was performed to confirm the consistency between the scale and theoretical structure [26, 27]. In the EFA, the eigenvalues using the Kaiser criterion, scree plot, parallel analysis, and theoretical basis of cultural beliefs were considered to select the optimal number of factors. After EFA was conducted using principal component analysis with a varimax rotation, CFA was performed using structural equation modeling (SEM) with oblique (intercorrelated) factors. The data were also examined based on correlations among variables, path analysis, parameter estimates, and the model’s fit to the observable data. Model fit was assessed by examining the relative chi-square (chi-square/degrees of freedom), root mean square error of approximation (RMSEA), goodness of fit index (GFI), standardized root mean square residual (SRMR), root mean square error of approximation (RMSEA), normed fit index (NFI), and comparative fit index (CFI). Factor loadings of each item were expected to be 0.40 or greater [28]. Reliability of all items in each scale was evaluated using Cronbach’s coefficient alpha. Each of the subscales was expected to have reliability with a Cronbach’s alpha of 0.70 or greater [29].

### Ethical considerations

The institutional review board at the Chosun University approved the protocols in the present study. All participants were informed in verbal or written form about the purpose and procedures of the study, estimated risks and benefits, privacy protection and confidentiality, as well as their right to freely withdraw from the study at any time.

## Results

### Sample characteristics

Questionnaires completed by 884 individuals were analyzed in the present study (328 men and 556 women). The average participant age was 60 years (M = 59.87; SD = 8.88; range 40–87). A total of 282 (31.9%) had a bachelors or higher degree, 767 (86.8%) were married, and 453 (51.3%) were employed.

### Construct validity

EFA analysis demonstrated that the data were suitable because the Kaiser-Meyer-Olkin (KMO) result was 0.93 and the chi-square value for Bartlett’s test of sphericity was 27,246.71 (*p* < 0.01). Using the EFA, four factors (factor 1: cancer fatalism; factor 2: health fatalism; factor 3: crisis health orientation and external control; and factor 4: preventive health orientation and internal control) were extracted and the latent root considered (eigenvalue > 1) based on eigenvalues > 1 using the Kaiser criterion, parallel analysis, scree plot, and theoretical basis (Table 2). Although the scree plot showed 4 clear changes in the slope, the eigenvalues (> 1) and parallel analysis using the Monte Carlo primary component analysis simulation resulted in more than 4 factors, but there is a single item under a factor as well as items with cross-factor loadings. The number of factors was reconsidered because (a) a single item is insufficient to measure a construct [30] and items loading on more than one factor are also suspect [31]; and (b) the number of factors that a parallel analysis produces may be an overestimate of the true dimensionality of the data [29]. Furthermore, it is important for the researcher to determine if the grouping of items in the EFA is theoretically consistent [31]. All evidence in the EFA was considered together for determining the number of factors underlying a set of item responses. The cumulative percentage of variance accounted for by these four factors was 48.12%. Although some items were moved to different factors, item factor loadings of the EFA ranged from 0.26 to 0.81. CFA using SEM was conducted with the four factors that were extracted from the EFA. Because the model did not fit the observed data, model respecification for all scales was needed. The results obtained from the CFA indicated that the model fit the data well, with adequate fit index values (Table 3). Although the fit index values in the present study and the chi-square results of three factors (factors 1, 2, and 3) had large values (*P* < 0.05), indicating that the model does not fit the data with large residual effects, other fit indices should be considered because chi-square statistics are inflated by large sample sizes [32]. The data comply with the theoretical structure of cultural beliefs (cancer fatalism, health fatalism, crisis health orientation and external control, preventive health orientation, and internal control) for the GFI, SRMR, RMSA, NFI, and CFI results. Based on the CFA, the factor loadings of items ranged from 0.21 to 0.92 (Table 4). Three items in the EFA and 11 items in the CFA had a factor loading < 0.40.

**Table 2:** Rotated exploratory factor analysis of the cultural belief scales

Factor 1: Cancer fatalism	Factor 2: Health fatalism	Factor 3: Crisis health orientation and external control	Factor 4: Preventive health orientation and internal control
CF1 0.53 CF2 0.67 CF3 0.61 CF4 0.71 CF5 0.72 CF6 0.73 CF7 0.69 CF8 0.47 CF9 0.74 CF10 0.62 CF11 0.76 CF12 0.77 CF13 0.79 CF14 0.68 CF15 0.69 CF16 0.47 CF17 0.31 CF18 0.50 HF11 0.46 HF12 0.39	HF1 0.50 HF2 0.59 HF3 0.72 HF4 0.75 HF5 0.78 HF6 0.75 HF7 0.68 HF8 0.69 HF9 0.66 HF10 0.58 EC9 0.40 EC10 0.44	CHO1 0.26 CHO2 0.51 CHO3 0.49 EC1 0.50 EC2 0.69 EC3 0.60 EC4 0.71 EC5 0.67 EC6 0.69 EC7 0.63 EC8 0.47	PHO1 0.80 PHO2 0.81 PHO3 0.79 PHO4 0.66 PHO5 0.46 IC1 0.73 IC2 0.71 IC3 0.60 IC4 0.41
Eigenvalue 14.60	5.11	2.75	2.58
Variance explained 28.07	9.82	5.28	4.95

*CF* cancer fatalism, *HF* health fatalism, *EC* external control, *CHO* crisis health orientation, *PHO* preventive health orientation, *IC* internal control

**Table 3:** Respecified measurement model fitness test results of the cultural belief subscales from confirmatory factor analysis

Fitness index	*χ*2 (*p*)	df	CMIN/DF	GFI	SRMR	RMSEA (90% CI)	NFI	CFI
Criteria	(> 0.05)		< 2	≥ .95	≤ .05	≤ .05	≥ .90	≥ .95
Cancer fatalism	220.70 (0.00)	112	1.75	0.98	0.03	0.03 (0.02–0.04)	0.98	0.99
Health fatalism	62.92 (0.00)	29	2.17	0.99	0.03	0.04 (0.02–0.05)	0.99	0.99
Crisis health orientation and external control	46.64 (0.01)	25	1.87	0.99	0.01	0.03 (0.02–0.05)	0.99	0.99
Preventive health orientation and internal control	20.25 (0.38)	19	1.07	0.99	0.01	0.01 (0.00–0.03)	0.99	1.00

*CMIN/DF* chi-square minimum/degree of freedom, *GFI* goodness of fit index, *SRMR* standardized root mean square residual, *RMSEA* root mean square error of approximation, *CI* confidence interval, *NFI* normed fit index, *CFI* comparative fit index

**Table 4:** Confirmatory factor analysis of the cultural belief scales

Scale and items	Corrected item-Total correlation	Factor loadings
Factor 1 (cancer fatalism)		
CF9. I think if someone is meant to have colorectal cancer, he/she will have colorectal cancer.	0.71	0.80
CF12. I think if someone has colorectal cancer and receives treatment for it, he/she will probably still die from the colorectal cancer.	0.74	0.80
CF13. I think if someone was meant to have colorectal cancer, he/she will get colorectal cancer regardless of what doctors and nurses tell him/her to do.	0.76	0.80
CF11. I think if someone gets colorectal cancer, he/she will die from it regardless of whether it is detected early or late.	0.72	0.76
CF6. I think if someone gets colorectal cancer, he/she will die soon.	0.70	0.75
CF15. I think colorectal cancer will kill me no matter when it is found and how it is treated.	0.67	0.75
CF7. I think if someone gets colorectal cancer, that’s the way he/she was meant to die.	0.70	0.74
CF5. I think if someone gets colorectal cancer, it was meant to be.	0.74	0.73
CF2. I think if someone has colorectal cancer, it is already too late to receive treatment.	0.68	0.72
CF4. I think if someone is meant to get colorectal cancer, he/she will get it regardless of what he/she does.	0.70	0.72
CF14. I think if someone is meant to have colorectal cancer, he/she will get bowel cancer regardless of whether he/she eats healthy foods.	0.66	0.65
CF10. I think some people do not want to know if they have colorectal cancer because they do not want to know they may be dying from it.	0.60	0.61
CF3. I think someone can eat fatty foods all of their life, and if he/she is not meant to get colorectal cancer, he/she won’t get it.	0.62	0.60
CF18. I think there is little one can do to prevent cancer.	0.56	0.54
CF1. I think if someone is meant to have colorectal cancer, he/she will get colorectal cancer despite the type of food he/she eats.	0.55	0.50
CF16. I think it is fate to get cancer.	0.56	0.50
CF8. I think getting checked for colorectal cancer makes people scared that they may really have colorectal cancer.	0.45	0.44
HF11. I often feel helpless in dealing with the problems of life.	0.50	0.42
CF17. I think cancer is always fatal.	0.34	0.28
HF12. There is really no way I can solve some of the problems I have.	0.41	0.29
Factor 2 (health fatalism)		
HF9. How long I live is a matter of luck.	0.74	0.92
HF8. I think destiny or fate is determined by God.	0.74	0.82
HF7. I think health or illness is determined by God.	0.72	0.75
HF6. I will die when I am fated to die.	0.71	0.73
HF10. I will stay healthy if I am lucky.	0.62	0.70
HF5. How long I live is predetermined.	0.75	0.67
HF4. I think health or illness is a matter of fate.	0.70	0.62
EC10. Luck has a lot to do with whether I am healthy or ill.	0.53	0.58
HF3. Life is predetermined.	0.66	0.55
EC9. It is solely up to God to decide if I am healthy or ill.	0.46	0.43
HF2. What will happen will happen regardless of what I do.	0.54	0.40
HF1. I cannot control life and death.	0.41	0.27
Factor 3 (crisis health orientation and external control)		
EC6. There is nothing that I can do to detect health problems early.	0.68	0.88
EC5. I have little influence over the things that happen to me.	0.62	0.80
EC7. There is nothing that I can do to detect colorectal cancer early.	0.59	0.79
EC4. Other powerful people decide when I should be screened for health problems.	0.61	0.62
EC2. Friends decide when I should be screened for health problems.	0.63	0.60
EC8. Identifying health problems early is a matter of chance.	0.47	0.58
EC3. Health care providers, such as the doctor, decide when I should be screened for health problems.	0.44	0.42
CHO2. Planning for regular health screenings is not important.	0.50	0.36
CHO3. As long as I am feeling well now, it is not important for me to have regular health screenings.	0.45	0.33
EC1. My family members decide when I should be screened for health problems.	0.37	0.21
CHO1. I only need to see my health care provider when I am sick.	0.25	0.16
Factor 4 (preventive health orientation and internal control)		
PHO1. Being healthy is important for my future.	0.69	0.92
PHO2. It makes sense to take care of my health now so I can be healthy in the future.	0.69	0.91
PHO3. It is important for me to do things now to prevent health problems.	0.68	0.86
IC1. I can make a difference in my health by detecting problems early.	0.62	0.62
PHO4. Identifying health problems early is important to me.	0.55	0.58
PHO5. It is important for me to plan to have CRC screening.	0.31	0.33
IC2. I should take it upon myself to find health problems early.	0.48	0.33
IC3. Finding health problems early is my responsibility.	0.52	0.33
IC4. I have a lot to do with finding health problems early.	0.47	0.31

*CF* cancer fatalism, *HF* health fatalism, *EC* external control, *CHO* crisis health orientation, *PHO* preventive health orientation, *IC* internal control

### Reliability

The reliability of the cultural belief subscales was assessed by Cronbach’s alpha coefficients, which are estimates of reliability (Table 5). All subscales demonstrated satisfactory reliability, with Cronbach’s alpha values > 0.70, according to the guidelines established by DeVellis [29].

**Table 5:** Internal consistency reliability of the cultural belief subscales

Scale	Number of items	Mean of items	Mean of item SDs	Cronbach’s alpha
Factor 1. Cancer fatalism	20	2.36	0.78	0.93
Factor 2. Health fatalism	12	2.50	1.05	0.90
Factor 3. Crisis health orientation and external control	11	2.23	0.86	0.82
Factor 4. Preventive health orientation and internal control	9	3.95	0.70	0.83

*SD* standard deviation

## Discussion

Although cultural beliefs influenced cancer screening behavior in Koreans [9–11, 14], culturally sensitive, reliable and valid instruments measuring cultural beliefs were not available for Koreans in the Republic of Korea. This is the first study that attempted to adapt and validate cultural belief scales for CRC screening in Koreans through several methodological steps. Individual interviews revealed that Koreans in the Republic of Korea had almost the same cultural beliefs regarding CRC screening as Korean Americans in the US [12]. Thus, the initial cultural belief scales were not changed significantly, except for the modification of words from unclear to clear and understandable and removal of redundancies from the sentences. The final cultural belief scales for CRC screening were culturally sensitive, reliable and valid for Koreans through the rigorous instrument adaptation and validation processes.

As shown in Table 2, EFA revealed that all items for cancer fatalism, including three cancer fatalism items that were adapted from the health fatalism scale (CF 16, 17, and 18), loaded on the cancer fatalism construct, but two health fatalism items (HF11 and 12) loaded on the cancer fatalism construct and two external control items (EC 9 and 10) loaded on the health fatalism construct. A feature of the cancer fatalism construct was helplessness or powerlessness due to negative health consequences [15]; thus, HF11 and 12 items related to helplessness could be indicators of cancer fatalism. Additionally, another feature of health fatalism was lack of control over external events [12]; thus, the EC9 and 10 items related to external control by God or luck could be indicators of health fatalism.

Although low total explained variance using a 4-factor model (48.12%) was reported in this study, methodological studies investigating belief scales about cancer screening have reported similar explained variance. For example, Lee et al. [15] examined the cultural belief scales among Korean Americans and conducted an exploratory factor analysis using all of the cultural belief items, resulting in 5 factors accounting for 46.55% of the variance. The five-factor model (cancer fatalism, health fatalism, health temporal orientation and internal control, external control, physical space) explained 7.28, 6.05, 4.89, 4.31, and 3.40% of the variance, respectively [15]. Rawl [33] also reported that explained variance for the 2-factor model factor analyses (benefits and barriers scales of fecal occult blood test, flexible sigmoidoscopy, colonoscopy) was 34, 33, and 33%, respectively. Champion [34] reported a 6-factor model (confidence, barriers, susceptibility, health motivation, seriousness, and barriers) for breast cancer screening behaviors, finding a total of 45.7% (16.7, 9.5, 8.0, 6.7, 6.2, and 4.6%, respectively). The low total variance of the model in this study may have resulted from cultural belief scales having many items (a total of 52 items) measuring multivariate variables. Items with low factor loadings were included in the model because the purpose of this study was to adapt and validate existing cultural belief scales for CRC screening among Koreans in the Republic of Korea.

Surprisingly, factor 3 consisted of items associated with crisis health orientation and external control, and factor 4 consisted of items associated with preventive health orientation and internal control, which may show that health temporal orientation and personal control were not separate but were intertwined in Koreans’ cognition and mind. Regarding factor 3, people who had crisis health orientation believed that symptoms are cues to seek health care. Thus, they did not go to medical facilities for cancer screening without having symptoms, but they would undergo cancer screening if external people, such as health care providers, encouraged them to undergo cancer screening. Similarly, regarding factor 4, people with a preventive health orientation believed that they should undergo cancer screening to detect health problems early. This result is similar to that in Lee et al.’s study [15], which tested cultural belief scales in Korean Americans, and health temporal orientation and internal control were loaded as one factor. Additionally, the CFA revealed that items with high factor loadings > 0.4 were related to the external control on factor 3 (Table 4). Similarly, most of the dominant factor 4 items with factor loadings > 0.4 were associated with the preventive health orientation.

The present study revealed significant implications through several methodological steps. First, the items in the EFA and CFA with factor loadings < 0.4 in the present study should be considered for removal from the scale. Findings from individual interviews were helpful for understanding relationships between poor-performing items and constructs. Regarding cancer fatalism, CF 17 “I think cancer is always fatal” performed poorly in both the EFA and CFA. Based on the interviews, Korean participants believed that cancer was not always fatal because cancer could be treatable when it was found early, which caused inconsistency between the item and construct. Furthermore, interestingly, both EFA and CFA showed that the item CHO 1, “I only need to see my health care provider when I am sick”, was poorly performed with respect to factor 3, which was in accordance with our previous study in Korean Americans [15]. CHO 1 may be unclear regarding whether to measure Koreans’ awareness or actual behavior. All of the participants in the individual interviews agreed that they should see health care providers when they did not have symptoms, but they did not actually see health care providers until they were sick. There were differences between Koreans’ awareness and actual behaviors regarding health care utilization. In this case, item contents should measure both awareness and actual behaviors in future practice and research. Second, traditional cultural beliefs, including crisis or preventive health orientation toward health care use, and internal or external control, were investigated in the present study. Theoretically, these beliefs are separate; however, in reality, these beliefs are intertwined in Koreans. Further research needs to examine traditional Korean cultural beliefs to increase the understanding of the relationships among these beliefs and cancer screening behaviors. Also, future research needs to refine instruments, retest reliability and validity, and finalize belief scales.

While the present study provided important findings regarding the cultural belief scales in Koreans, we advise caution in interpretation of the findings due to research limitations. First, limitations regarding generalizability of the study results should be acknowledged due to the use of a convenience sample of Koreans who resided in a metropolitan city in the Republic of Korea. Second, the present study examined only EFA and CFA; thus, other validation methods for cultural belief scales need to be investigated to examine them more accurately.

## Conclusion

This is the first study that adapted and validated theory-based cultural belief scales regarding CRC screening among Koreans. The results of the present study showed that four-factor cultural belief scales were culturally sensitive, reliable and valid for Koreans. The final cultural belief scales could be used to identify cultural beliefs more accurately and specifically, as well as to develop effective interventions to increase CRC screening behaviors among Koreans in the Republic of Korea.

## Additional file

Additional file 1: English version of the cultural belief scales about colorectal cancer screening. (DOCX 31 kb)

## Abbreviations

CF: Cancer fatalism
CFA: Confirmatory factor analysis
CFI: Comparative fit index
CHO: Crisis health orientation
CI: Confidence interval
CMIN/DF: Chi-square minimum/degree of freedom
CRC: Colorectal cancer
EC: External control
EFA: Exploratory factor analysis
GFI: Goodness of fit index
HF: Health fatalism
IC: Internal control
NFI: Normed fit index
PHO: Preventive health orientation
RMSEA: Root mean square error of approximation
SEM: Structural equation modeling
SRMR: Standardized root mean square residual
US: United States
