# Supplementary material for: Factor structure and internal reliability of cultural belief scales about colorectal cancer screening among Koreans in the Republic of Korea
Source: BMC Public Health. 2018 Nov 29;18:1328. doi: 10.1186/s12889-018-6240-9 (PMC6267900; doi:10.1186/s12889-018-6240-9)
Supplement: Supplementary file 1 — English version of the cultural belief scales about colorectal cancer screening. (DOCX 31 kb) [file 12889_2018_6240_MOESM1_ESM.docx]

English version of the cultural belief scales about colorectal cancer screening

| Scale and items |
| --- |
| **Cancer fatalism**  CF1. I think if someone is meant to have colorectal cancer, he/she will get colorectal cancer despite the type of food he/she eats. |
| CF2. I think if someone has colorectal cancer, it is already too late to receive treatment. |
| CF3. I think someone can eat fatty foods all of their life, and if he/she is not meant to get colorectal cancer, he/she won't get it. |
| CF4. I think if someone is meant to get colorectal cancer, he/she will get it regardless of what he/she does. |
| CF5. I think if someone gets colorectal cancer, it was meant to be. |
| CF6. I think if someone gets colorectal cancer, he/she will die soon. |
| CF7. I think if someone gets colorectal cancer, that's the way he/she was meant to die. |
| CF8. I think getting checked for colorectal cancer makes people scared that they may really have colorectal cancer. |
| CF9. I think if someone is meant to have colorectal cancer, he/she will have colorectal cancer. |
| CF10. I think some people do not want to know if they have colorectal cancer because they do not want to know they may be dying from it. |
| CF11. I think if someone gets colorectal cancer, he/she will die from it regardless of whether it is detected early or late. |
| CF12. I think if someone has colorectal cancer and receives treatment for it, he/she will probably still die from the colorectal cancer. |
| CF13. I think if someone was meant to have colorectal cancer, he/she will get colorectal cancer regardless of what doctors and nurses tell him/her to do. |
| CF14. I think if someone is meant to have colorectal cancer, he/she will get bowel cancer regardless of whether he/she eats healthy foods. |
| CF15. I think colorectal cancer will kill me no matter when it is found and how it is treated.  CF16. I think it is fate to get cancer.  CF17. I think cancer is always fatal.  CF18. I think there is little one can do to prevent cancer. |
|  |
| **Health fatalism**  HF1. I cannot control life and death. |
| HF2. What will happen will happen regardless of what I do. |
| HF3. Life is predetermined. |
| HF4. I think health or illness is a matter of fate. |
| HF5. How long I live is predetermined. |
| HF6. I will die when I am fated to die. |
| HF7. I think health or illness is determined by God. |
| HF8. I think destiny or fate is determined by God. |
| HF9. How long I live is a matter of luck. |
| HF10. I will stay healthy if I am lucky. |
| HF11. I often feel helpless in dealing with the problems of life. |
| HF12. There is really no way I can solve some of the problems I have. |
|  |
| **Health temporal orientation (crisis and preventive health orientation)** |
| CHO1. I only need to see my health care provider when I am sick. |
| CHO2. Planning for regular health screenings is not important. |
| CHO3. As long as I am feeling well now, it is not important for me to have regular health screenings. |
| PHO1. Being healthy is important for my future. |
| PHO2. It makes sense to take care of my health now so I can be healthy in the future. |
| PHO3. It is important for me to do things now to prevent health problems. |
| PHO4. Identifying health problems early is important to me. |
| PHO5. It is important for me to plan to have CRC screening. |
|  |
| **Personal control (internal and external control)** |
| IC1. I can make a difference in my health by detecting problems early. |
| IC2. I should take it upon myself to find health problems early. |
| IC3. Finding health problems early is my responsibility. |
| IC4. I have a lot to do with finding health problems early. |
| EC1. My family members decide when I should be screened for health problems. |
| EC2. Friends decide when I should be screened for health problems. |
| EC3. Health care providers, such as the doctor, decide when I should be screened for health problems. |
| EC4. Other powerful people decide when I should be screened for health problems. |
| EC5. I have little influence over the things that happen to me. |
| EC6. There is nothing that I can do to detect health problems early. |
| EC7. There is nothing that I can do to detect colorectal cancer early. |
| EC8. Identifying health problems early is a matter of chance. |
| EC9. It is solely up to God to decide if I am healthy or ill. |
| EC10. Luck has a lot to do with whether I am healthy or ill. |

*CF* cancer fatalism, *HF* health fatalism, *CHO* crisis health orientation, *PHO* preventive health orientation, *IC* internal control, *EC* external control,
